# Genetic insights into across pancreatitis types: the causal influence of immunoglobulin G N-glycosylation variants on disease risk

**DOI:** 10.3389/fimmu.2024.1326370

**Published:** 2024-03-18

**Authors:** Yulin Chen, Xue Li, Ran Lu, Yinchun Lv, Junman Ye, Qiaorong Huang, Wentong Meng, Feiwu Long, Jonas Burman, Xianming Mo, Chuanwen Fan

**Affiliations:** ^1^ West China Center of Excellence for Pancreatitis, Institute of Integrated Traditional Chinese and Western Medicine, Laboratory of Stem Cell Biology, State Key Laboratory, West China Hospital, Sichuan University, Chengdu, China; ^2^ Department of Occupational and Environmental Health, West China School of Public Health and West China Fourth Hospital, Sichuan University, Chengdu, China; ^3^ West China-PUMC C. C. Chen Institute of Health, West China School of Public Health, and West China Fourth Hospital, Sichuan University, Chengdu, China; ^4^ Department of Gastrointestinal, Bariatric and Metabolic Surgery, Research Center for Nutrition, Metabolism and Food Safety, West China-PUMC C.C. Chen Institute of Health, West China School of Public Health and West China Fourth Hospital, Sichuan University, Chengdu, China; ^5^ Department of Oncology and Department of Biomedical and Clinical Sciences, Linköping University, Linköping, Sweden

**Keywords:** IgG, N-glycosylation, pancreatitis, Mendelian randomization (MR), FinnGen, UK Biobank

## Abstract

**Background:**

While a few case-control studies indicated a possible correlation of IgG N-glycosylation patterns with pancreatitis, their restricted sample sizes and methodologies prevented conclusive insights into causality or distinguishing traits across pancreatitis types.

**Method:**

We conducted a two-sample Mendelian Randomization (MR) analysis to investigate the causal relationship between 77 IgG N-glycosylation traits and various types of pancreatitis, including acute pancreatitis (AP), chronic pancreatitis (CP), alcohol acute pancreatitis (AAP), and alcohol chronic pancreatitis (ACP). This analysis utilized summary-level data from genome-wide association studies (GWAS), employing methods such as IVW, MR-Egger, and weighted median. To ensure the robustness of our findings, several sensitivity analyses, including Cochran’s Q statistic, leave-one-out, MR-Egger intercept, and MR-PRESSO global test were conducted.

**Result:**

Our study uncovered the causal relationship between specific IgG N-glycosylation traits and various types of pancreatitis. Notably, an increase in genetically predicted IGP7 levels was associated with a decreased risk of developing AP. For CP, our data suggested a protective effect associated with higher levels of both IGP7 and IGP31, contrasting with increased levels of IGP27 and IGP65, which were linked to a heightened risk. Moreover, in the case of AAP, elevated IGP31 levels were causatively associated with a lower incidence, while higher IGP26 levels correlated with an increased risk for ACP.

**Conclusion:**

This study establishes causal relationship between specific IgG N-glycosylation patterns and varying risks of different pancreatitis forms, underscoring their potential as predictive biomarkers. These findings necessitate further exploration into the underlying mechanisms, promising to inform more personalized diagnostic and therapeutic strategies in pancreatitis management.

## 1 Introduction

Acute pancreatitis, a prevalent inflammatory disorder originating in the pancreas, triggers a systemic inflammatory response and presents varied clinical outcomes (1, 2). While the majority of cases are mild, approximately 20% progress to severe, developing complications (3, 4), and 8% evolve into chronic pancreatitis, thereby increasing the risk of metabolic disorders and pancreatic cancer (5).

The balance between pro-inflammatory and anti-inflammatory responses significantly influences the progression and outcomes of acute pancreatitis (6). A well-regulated inflammatory response is essential for restoring homeostasis. However, persistent, uncontrolled inflammation can exacerbate acute pancreatitis and potentially lead to chronic pancreatitis (7). Research has long been focused on the role of innate immune responses in acute pancreatitis (8, 9), but increasing findings highlight the equally critical role of adaptive immune cells, including T and B lymphocytes, in modulating the immune response and exacerbating the condition (9–13). Early overactivity of these cells can precipitate severe complications in acute pancreatitis, such as multiple organ dysfunction syndrome (14). Regulatory T and B lymphocytes, vital for controlling inflammation and promoting immune tolerance, show a marked decrease in severe acute pancreatitis compared to milder forms (15), suggesting their involvement in the disease’s progression and potential transition to chronic pancreatitis. A noteworthy aspect of severe acute pancreatitis is the persistent reduction in B cell counts, indicating a prolonged immune disruption (12). This observation aligns with the research indicating that the crucial roles of B lymphocytes in the development and progression of both acute and chronic pancreatitis (13, 16–18).The differences in B lymphocyte counts have been observed between mild and severe cases of acute pancreatitis, with an increase in B lymphocyte counts being associated with a higher risk of organ failure associated with pancreatitis (19). Additionally, significant alterations in the production of immunoglobulins, crucial molecules synthesized by B lymphocytes, have been noted in pancreatitis patients compared to healthy individuals. Specifically, levels of serum Immunoglobulin M and Immunoglobulin G (IgG) are significantly decreased in pancreatitis patients with infectious complications, with reduced IgG levels being particularly evident in fatal cases (9). This highlights the crucial immunomodulatory role of IgG during the development of pancreatitis.

The immunomodulatory role of IgG is further influenced by the N-glycosylation at the conserved asparagine 297 within its fragment crystallizable (Fc) region, acting as a critical immunoregulatory switch (20). The process of adding N-glycans to the Fc region of antibodies significantly influences their interaction with the immune system, affecting how they activate various immune responses. For example, removing fucose, a process known as afucosylation, enhances the antibody’s ability to bind to the FcγRIIIa receptor, thereby increasing its capacity for antibody-dependent cellular cytotoxicity (ADCC), a critical mechanism for targeting and destroying harmful cells. Additionally, introducing a bisecting N-acetylglucosamine (GlcNAc), or bisection, is thought to potentially amplify ADCC activity (21–24), despite ongoing discussions about its precise impact on the Fc region’s role in diseases and inflammation (25). On the other hand, the presence or absence of galactose (galactosylation) is crucial, as its lack is associated with triggering autoimmunity and inflammation. Conversely, adding sialic acid (sialylation) tends to have an anti-inflammatory effect and extends the lifespan of antibodies in the bloodstream, aiding in a more regulated immune response (26). Changes in these glycan structures, such as increased levels of antibodies lacking galactose (agalactosylated) or sialic acid (asialylated), often correlate with more severe disease states, particularly in autoimmune diseases where decreased galactosylation and sialylation are linked to exacerbated conditions (27, 28). Notably, distinct IgG-glycosylation profiling has been identified in patients with autoimmune pancreatitis and pancreatic cancer (29). Furthermore, alterations in the N-glycosylation profiles of plasma proteins, including IgG and other antibodies, have been documented during conditions such as sepsis and acute pancreatitis (6). These findings indicate the existence of distinct IgG-glycosylation profiles across various types of pancreatitis. However, comprehensive data on IgG-glycosylation traits across different forms of pancreatitis are scarce, and the specific roles and causal relationships between IgG-glycosylation traits and various forms of pancreatitis, including acute pancreatitis (AP), chronic pancreatitis (CP), alcohol acute pancreatitis (AAP), and alcohol chronic pancreatitis (ACP), remain to be elucidated.

To bridge this knowledge gap, we have employed Mendelian Randomization (MR) analysis, using genetic variants as instrumental variables (IVs), to explore potential causal relationships between IgG-glycosylation traits and various forms of pancreatitis in a comprehensive population. Furthermore, this study is designed to identify unique IgG N-glycosylation profiles across pancreatitis categories, thereby shedding light on the complex interplay between IgG glycosylation and the pathogenesis of pancreatitis.

## 2 Method and material

### 2.1 Ethics statement

In this study, we utilized publicly available summary statistics for both either IgG N-glycosylation and pancreatitis, without involving any original data collection. The consortia that provided these statistics had previously obtained ethical approval and informed consent from all participants, as documented in their respective publications (30, 31).

### 2.2 Data sources for IgG N-glycosylation

We sourced the summary-level GWAS data for IgG N-glycosylation traits from a comprehensive meta-analysis involving 8090 individuals of European ancestry. This dataset covered 77 IgG N-glycosylation traits (IGP1-77), including 23 directly measured N-glycosylation traits (IGP1-23) and 54 derived N-glycosylation traits (IGP24-77), as detailed in **Supplementary Table S1**. The 23 directly measured traits were quantified using ultraperformance liquid chromatography (UPLC), each corresponding to a UPLC peak indicative of a major biantennary complex N-glycan structure. These complex structures were characterized by specific features, including core-fucose, bisecting N-acetylglucosamine (GlcNAc), terminal galactose, and terminal sialic acids present on the antennae. The 54 derived traits calculated from these direct measurements represent relative abundances or proportions of specific glycan groups, categorized defined by structural similarities.

### 2.3 Data source for AAP, ACP, AP, and CP

For summary statistics for AP, we utilized data from a GWAS meta-analysis by Bourgault, et al, which incorporated data from the UK Biobank, the Estonian Biobank, and FinnGen (30). This analysis involved 10,630 AP cases and 844,679 controls, examining 9,570,209 SNPs with a minor allele frequency of ≥0.01 across participants of European ancestry. For AAP, ACP, and CP, summary statistics were derived from R9 release of the FinnGen consortium (https://storage.googleapis.com/finngen-public-data-r9/summary_stats), comprising 931 AAP cases with 376,346 controls, 1,794 ACP cases with 375,483 controls, and 3,320 CP cases with 330,903 controls.

### 2.4 Selection of IVs for IgG N-glycosylation traits

SNPs associated with IgG N-glycosylation traits that achieved genome-wide significance (p < 5E-8) were selected as potential IVs, based on summary statistics for IgG N-glycosylation as previously described. To ensure the analysis was based on representative genetic signals, any SNPs found in close linkage disequilibrium (LD, r^2^ < 0.001 within a 10 Mb range) were removed. The R^2^ and F statistics for each potential IV were calculated using the established formulas: R^2 = ^2 × EAF × (1−EAF) × β^2^ and F statistic = R^2^ × (N−2)/(1−R^2^). Any SNP with an F statistic below 10 was excluded to minimize the risk of weak instrument bias. To further refine the IVs, PhenoScanner V2 (http://www.phenoscanner.medschl.cam.ac.uk/), an online tool, was used to filter out SNPs associated with pancreatitis. Additionally, IgG N-glycosylation traits represented by fewer than 2 SNPs after the aforementioned filtering were discarded. This thorough process of selection and filtration led us to identify and focus on 63 IgG N-glycosylation traits for this analysis.

### 2.5 MR analysis

We estimated the causal associations of IgG N-glycosylation traits with AP, CP, AAP, and ACP using three two-sample MR methods: random-effects inverse-variance-weighted (IVW), MR-Egger, and weighted median. The IVW method was primarily employed to derive the outcomes. To assess the robustness of these outcomes, we also applied the MR-Egger and weighted median methods. We presented the effect estimates as odds ratios (ORs) with 95% confidence intervals (CIs), reflecting the impact on pancreatitis outcomes per genetically predicted increase in IgG N-glycosylation levels. Considering the multiple tests conducted, we adjusted the significance level thresholds using the Benjamini-Hochberg method, defining significance as an FDR of less than 5%.

### 2.6 The MR sensitivity analysis

We performed Cochran’s Q statistic and leave-one-out analysis to evaluate the heterogeneity across each SNP. Additionally, the MR-Egger intercept test and the MR-PRESSO global test were conducted to detect any presence of horizontal pleiotropy. All these analyses were executed using TwoSampleMR (version 0.5.6) and MR-PRESSO (version 1.0) packages in R software (version 4.2.0).

## 3 Result

### 3.1 The selection of IgG N-glycosylation genetic instruments

To investigate the causal effect of IgG N-glycosylation traits on pancreatitis, we selected genetic variants significantly associated with these traits as IVs (**Supplementary Table S2**). This selection was based on the most comprehensive GWAS meta-analysis available, encompassing data from 8,090 European individuals. Our analysis included 304 independent SNPs representing 63 IgG N-glycosylation traits. Notably, these SNPs were not associated with the pancreatitis (**Supplementary Tables S4**–**S7**). Among these traits, the number of IVs for each IgG N-glycosylation trait ranged up to eight, with a median number of six IVs across the 63 traits (**Supplementary Table S2**). The F-statistics for the chosen SNPs, which ranged from 33 to 1480, indicated robust effects, avoiding weak instrument bias (**Supplementary Table S3**).

### 3.2 Estimated causal association of IgG N-glycosylation traits with AP

**Figure 1:**
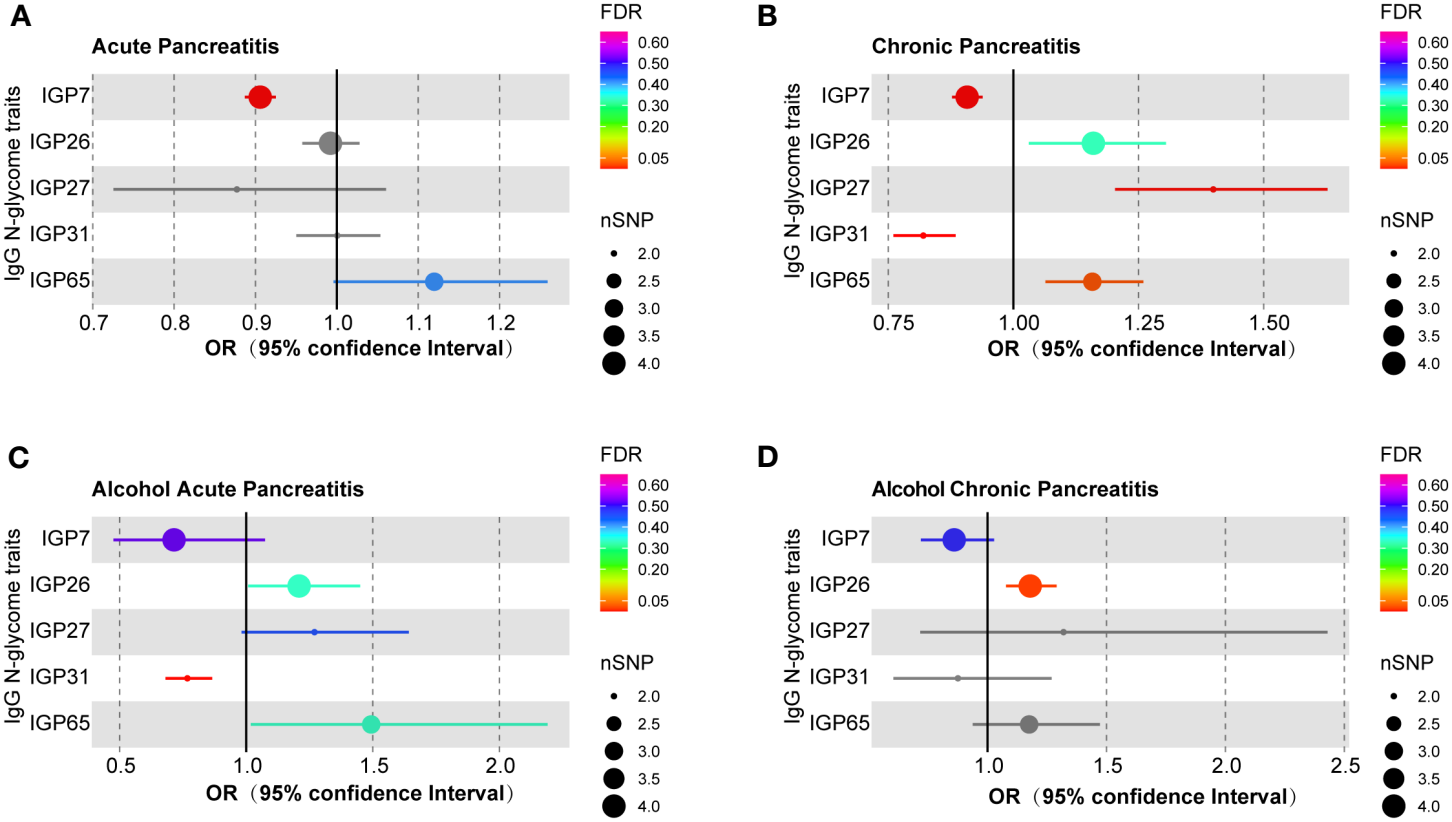
MR estimates for the association of IgG N-glycosylation traits with different pancreatitis types. **(A)** The casual effect of IgG N-glycosylation traits on **(A)** AP, **(B)** CP, **(C)** AAP, and **(D)** ACP. The OR was estimated using the random-effects inverse variance weighted method. MR, Mendelian Randomization; SNPs, single-nucleotide polymorphisms; OR, odds ratio.

After multiple testing corrections, a strong causal relationship was observed between the genetically predicted IgG N-glycosylation trait IGP7 and AP, with increased IGP7 correlating with reduced risk of AP (OR = 0.906, 95% CI = 0.887-0.925, FDR = 5.13E-18) (**Figure 1A**; **Supplementary Table S8**). This result was consistent across MR Egger and weighted median models (**Supplementary Table S9**). The remaining 62 IgG N-glycosylation traits showed no causal association with AP (**Supplementary Table S8**). Further sensitivity analyses indicated no SNP heterogeneity per Cochran’s Q test and leave-one-out analysis (**Supplementary Tables S10**, **S11**). The horizontal pleiotropy was ruled out as per MR-Egger and MR-PRESSO analysis. (**Supplementary Tables S12**, **S13**).

### 3.3 Estimated causal association of IgG N-glycosylation traits with CP

Genetically predicted IgG N-glycosylation traits IGP7 (OR = 0.908, 95% CI = 0.878-0.939, FDR = 1.80E-6) and IGP31 (OR = 0.820, 95% CI = 0.760-0.885, FDR = 2.40E-5) were causally correlated with a decreased CP risk (**Figure 1B**; **Supplementary Table S8**). Conversely, IGP27 (OR = 1.399, 95% CI = 1.203-1.627, FDR = 7.69E-4) and IGP65 (OR = 1.158, 95% CI = 1.064-1.259, FDR = 0.024) were associated with an increased CP risk (**Figure 1B**; **Supplementary Table S8**). These findings were consistent in MR Egger and weighted median models (**Supplementary Table S9**). However, no causal links were found between IGP31 and either AP or ACP, and neither for IGP27 or IGP65 with AP, AAP, or ACP (**Supplementary Table S8**). Cochran’s Q test results indicated no SNP heterogeneity, though minor heterogeneity was noted for IGP27 (*P* = 0.047) and IGP31 (*P* = 0.034) in leave-one-out analysis (**Supplementary Tables S14**, **S15**). Meanwhile, these associations persisted in MR-PRESSO corrected results. No evidence of horizontal pleiotropy was found using MR-Egger intercepts and MR-PRESSO methods (**Supplementary Tables S16**, **S17**).

### 3.4 Estimated causal association of IgG N-glycosylation traits with AAP

The genetically predicted IgG N-glycosylation trait IGP31 was causally associated with a reduced AAP risk (OR = 0.768, 95% CI = 0.681-0.865, FDR = 7.69E-4) (**Figure 1C**; **Supplementary Table S8**). These results were consistent with MR Egger and weighted median models (**Supplementary Table S9**). However, no causal associations were observed for IGP31 with ACP or AP (**Supplementary Table S8**). Furthermore, we detected no heterogeneity among the SNPs (**Supplementary Table S18**), and the leave-one-out analysis (**Supplementary Table S19**) confirmed this consistency, revealing that no single SNP influenced the overall results. Additionally, neither the MR-Egger nor the MR-PRESSO methods showed signs of horizontal pleiotropy (**Supplementary Tables S20**, **S21**).

### 3.5 Estimated causal association of IgG N-glycosylation traits with ACP

The genetic prediction of IgG N-glycosylation trait IGP26 (OR = 1.179, 95% CI = 1.078-1.290, FDR = 0.014) was found to be causally associated with an increased risk of ACP (**Figure 1D**; **Supplementary Table S8**). This association was validated by analysis using MR Egger and weighted median models (**Supplementary Table S9**). However, there were no observed causal relationships between IGP26 and either AP or CP (**Supplementary Table S8**). Further, our sensitivity analysis, including Cochran’s Q test (**Supplementary Table S22**) and leave-one-out analysis (**Supplementary Table S23**), did not reveal any significant heterogeneity among the SNPs. Also, we found no evidence of horizontal pleiotropy for IgG N-glycosylation traits when using MR-Egger and MR-PRESSO methods (**Supplementary Tables S24**, **S25**).

## 4 Discussion

Uncovering the causal effects of genetically predicted IgG N-glycosylation traits on pancreatitis is crucial for advancing our understanding of their role in this condition. This study is the first to explore the causal associations of these traits with both acute and chronic pancreatitis, including AP, CP, AAP, and ACP, in European populations. Our findings reveal that an increase in genetically predicted IGP7, which directly measures IgG N-glycosylation trait, appears to protective against AP. This aligns with previous study identifying a similar correlation in scenarios of acute inflammation, such as early phase of sepsis and AP (6). However, our study delves deeper into the potential causal relationship between IGP7 and AP, extending the protective effect with CP. Considering that approximately 8% of acute pancreatitis cases may progress to CP (3, 32), the observed protective effect of IGP7 across both pancreatitis types underscores its significant role not only in the initial phase of inflammation but also in the transition from acute to chronic inflammation. This protective role of IGP7 extends beyond the early stages of the disease and potentially influenced the progression trajectory. However, the specific mechanisms underlying this protective effect require further elucidation through experimental studies. Additionally, a protective effect against CP was observed from IGP31, a trait indicative of the proportion of monosialylated, fucosylated, and digalactosylated structures with bisecting GlcNAc within total IgG glycans. This trait, reflecting overall IgG glycans, showed a protective effect similar to that of IGP7, underscoring the potential importance of specific IgG N-glycosylation structures in inflammation modulation.

Furthermore, we discovered significant associations between the IgG N-glycosylation traits IGP27 and IGP65 and an increased risk of CP, a finding not observed for AP. This presents a novel area of investigation, as current literature does not directly link these glycosylation traits with CP. The absence of direct evidence compels us to delve into the potential mechanisms through analyzing the characteristics of similarities and differences between IGP27 and IGP65. IGP27 is noted for its sialylation of all fucosylated structures including bisecting GlcNAc, while IGP65 is defined on the fucosylation of digalactosylated structures, specifically excluding bisecting GlcNAc. Their different structures suggest that fucosylation patterns in IgG N-glycans may significantly influence CP risk, rather than whether bisecting GlcNAc is present or not. Although the role of bisecting GlcNAc in CP is still unclear, its contrasting relevance in other chronic inflammation diseases has been documented. For instance, bisecting GlcNAc was upregulated in systemic lupus erythematosus (33), contrasting with rheumatoid arthritis (34), where bisecting GlcNAc appeared to exert minimal or no effect, and a downregulation was noted in granulomatosis with polyangiitis patients (35). These variations highlight the complex role of bisecting GlcNAc across different inflammation conditions. In contrast, our findings that fucosylation patterns, as shared by IGP27 and IGP65, were significantly associated with an increased risk of CP, suggesting an important role for these glycosylation traits in the pathogenesis of CP. This is supported by research in autoimmune pancreatitis, where significantly increased IgG1 fucosylation were observed, highlighting the unique impact of fucosylated structures in pancreatitis (29). Furthermore, a study on COVID-19 patients revealed an association with increased total IgG fucosylation with severity of COVID-19 (36). However, the presence of fucosylated IgG N-glycans is typically linked to reduced inflammation, attributed to a diminished capacity to mediate antibody-dependent cellular cytotoxicity (ADCC) (37–39). These variations in IgG N-glycan fucosylation may be influenced by the type and progression of the disease. Alternatively, these differences in IgG N-glycan fucosylation may be associated with different molecular mechanisms involved in the immune response. While fucosylated IgG N-glycans are known to contribute to anti-inflammatory processes and inflammation resolution, an imbalance in fucosylation could disrupt these processes. Such an imbalance may lead to prolonged or exacerbated inflammation, a characteristic feature of CP. Similarly, increased IgG N-glycan fucosylation might affect the formation and clearance of immune complexes (40). Alterations in glycosylation patterns can influence the solubility and clearance rates of these complexes, potentially leading to their accumulation and deposition (41). This process can promote inflammation in various tissues, including the pancreas (42). This complex interplay between IgG N-glycan fucosylation patterns and immune responses underscores the need for further investigation into how these mechanisms contribute to the pathogenesis and progression of CP.

Given the reported incidence of acute alcoholic pancreatitis as a leading cause in the European population (43), we further evaluated the correlation between IgG N-glycosylation traits and AAP or ACP. Intriguingly, akin to CP, IGP31 showed a negative causal association with AAP, but not with ACP. This parallel influence of IGP31 in both CP and AAP could be interpreted as an indication that acute alcoholic pancreatitis was frequently correlated with prolonged alcohol misuse. Even in the absence of overt pancreatitis symptoms, pancreatic tissue may undergo chronic microscopic inflammation and structural changes due to alcohol’s direct toxicity, impacting pancreatic acinar and stellate cells, altering pancreatic secretions, and affecting blood flow (44). Consequently, the pancreas of long-term alcohol consumers might already be in a compromised state, and thus prone to acute inflammation. Nevertheless, the predicted increase IgG N-glycosylation, including IGP31, did not exhibit a protective effect against ACP. In contrast, predicted increased IgG N-glycosylation IGP26, which measured the percentage of sialylation of all fucosylated structures without bisecting GlcNAc in total IgG glycans, was specifically associated with a highly risk of ACP. Although no studies have reported evidence of differences in IgG N-glycosylation traits between CP and ACP, recent studies have shown differences in the innate and adaptive immune responses among pancreatitis subtypes (45). Taken together with our results, this suggests that differences in immunopathogenic mechanisms of different subtypes of chronic pancreatitis.

Pro- and anti-inflammatory properties of IgG, which are pivotal in the pathology of various pancreatitis subtypes, were intricately influenced by N-glycosylation modifications of such as galactose, fucose, sialic acid, and bisecting GlcNAc (21, 25, 46). Concurrently, our current insights revealed that while different pancreatitis subtypes share certain IgG N-glycosylation patterns, they also present unique characteristics. These commonalities in IgG N-glycosylation may reflect a delicate equilibrium between the pro- and anti-inflammatory properties of IgG. Additionally, they could be tied to the homeostasis maintained by pancreatic exocrine cells and the surrounding microenvironment. This nuanced understanding underscored the complexity of inflammatory regulation during the occurrence and outcome of pancreatitis and highlighted the need for a more granular approach to its study, particularly in the context of immune responses and cellular environment interactions.

However, it’s crucial to note several limitations within our study. First, despite our analysis to assess the causal relationship between IgG N-glycosylation traits and various forms of pancreatitis, participants of our study only included European ancestry. This fact potentially narrows the generalizability of our findings to individuals from diverse ethnic backgrounds, such as those of Asian and African ancestry. Second, the absence of granular individual-level data from GWAS and clinical data on pancreatitis constrains our ability to conduct an in-depth analysis of the association between IgG N-glycosylation profiles and various aspects of pancreatitis. This includes its severity, etiology (such as gallstone-related or autoimmunity-related), and other clinical features, that cannot be further performed. Third, a significant limitation of our study is the small number of cases involving acute alcoholic pancreatitis and chronic pancreatitis. Studies with larger sample sizes are necessary for further validation. Fourth, the discussion on the negative impacts of IgG glycosylation traits on AAP and ACP is limited due to the lack of relevant research, small sample sizes of these subtypes, and the inherent variability in IgG N-glycosylation patterns. Lastly, to solidify the causal effects of IgG N-glycosylation traits in pancreatitis, and to explore the intricate mechanisms involved, additional *in vitro* and *in vivo* studies are imperative.

## 5 Conclusions

Overall, the current study establishes causal associations between specific IgG N-glycosylation patterns and varying risks of different pancreatitis forms, underscoring their potential as predictive biomarkers. These findings necessitate further exploration into the underlying mechanisms, promising to inform more personalized diagnostic and therapeutic strategies in pancreatitis management.

## Data availability statement

All the GWAS summary-level data used in our study are openly available online. Specifically, data on IgG N-glycosylation traits, as comprehensively analyzed by Klaric et al. (2020), can be found at https://datashare.ed.ac.uk/handle/10283/3238. For AP traits, we used summary statistics from a meta-analysis conducted by Bourgault et al. (2023), which pooled data from three significant research cohorts: the UK Biobank, the Estonian Biobank, and FinnGen. This information is available at http://ftp.ebi.ac.uk/pub/databases/gwas/summary_statistics/GCST90255001-GCST90256000/GCST90255375/. Additionally, we accessed summary statistics for AAP, ACP, and CP from the FinnGen consortium’s R9 release, which is openly accessible at https://storage.googleapis.com/finngen-public-data-r9/summary_stats. The comprehensive availability of this data supports the transparency and reproducibility of our findings, providing a solid foundation for our analyses.

## Author contributions

YC: Conceptualization, Data curation, Formal analysis, Investigation, Methodology, Project administration, Resources, Software, Validation, Visualization, Writing – original draft, Supervision, Writing – review & editing. XL: Conceptualization, Data curation, Formal analysis, Methodology, Resources, Software, Validation, Visualization, Writing – original draft, Investigation, Project administration, Supervision, Writing – review & editing. RL: Conceptualization, Data curation, Formal analysis, Methodology, Resources, Validation, Writing – review & editing. YL: Conceptualization, Data curation, Resources, Software, Writing – review & editing. JY: Conceptualization, Data curation, Resources, Writing – review & editing. QH: Conceptualization, Data curation, Formal analysis, Resources, Writing – review & editing. WM: Conceptualization, Data curation, Formal analysis, Resources, Software, Writing – review & editing. FL: Conceptualization, Data curation, Formal analysis, Resources, Writing – review & editing. JB: Writing – review & editing. XM: Funding acquisition, Project administration, Supervision, Validation, Writing – review & editing, Writing – original draft. CF: Funding acquisition, Project administration, Supervision, Validation, Writing – review & editing, Writing – original draft.
